# Aspirin at 120: Retiring, recombining, or repurposing?

**DOI:** 10.1002/rth2.12516

**Published:** 2021-05-28

**Authors:** Carlo Patrono, Bianca Rocca

**Affiliations:** ^1^ Department of Safety Section of Pharmacology Catholic University School of Medicine Rome Italy

**Keywords:** aspirin, cardiovascular disease, colorectal cancer, nonsteroidal anti‐inflammatory drugs, oral anticoagulants, P2Y_12_ inhibitors

## Abstract

During the past 20 years, we have witnessed the following trends in aspirin usage: (i) a “dropping” trend, characterized by the early discontinuation of low‐dose aspirin from dual antiplatelet therapy or triple antithrombotic therapy (oral anticoagulation plus dual antiplatelet therapy in patients with atrial fibrillation) following an acute coronary syndrome or after percutaneous coronary intervention; (ii) a “combinatorial” trend, featuring the addition of a lower dose of a P2Y_12_ inhibitor or direct oral anticoagulant drug to low‐dose aspirin for the long‐term treatment of stable patients with atherosclerotic cardiovascular disease; and (iii) a “repurposing” trend, characterized by growing interest in the oncologic community to assess the chemopreventive effect of aspirin against certain types of cancers (particularly of the gastrointestinal tract), both as primary prevention and adjuvant therapy.

The aim of this review is to present the mechanistic rationale underlying these trends, discuss the design and findings of trials testing novel treatments or new therapeutic applications of aspirin, and report on the ISTH Congress results on this topic.

**Essentials**

- Recently, low‐dose aspirin use has experienced dropping, combining, or repurposing trends.
- No convincing evidence justifies dropping aspirin rather than a P2Y_12_ inhibitor from dual or triple antiplatelet therapy.
- An aspirin/factor Xa inhibitor combination represents a valid approach to high‐risk secondary prevention.
- There is convincing evidence supporting a chemopreventive effect of aspirin against gastrointestinal cancers.

​

​

## 1 INTRODUCTION

**FIGURE 1:**
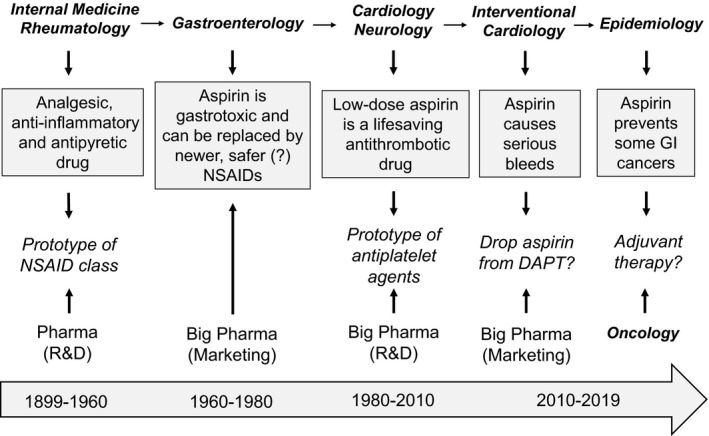
One hundred twenty years of aspirin‐inspired research and development. The figure schematically summarizes the three phases of aspirin development: (A) as an analgesic, antipyretic, and anti‐inflammatory agent; (B) as an antiplatelet drug; and (C) as a chemopreventive agent. Aspirin has inspired research throughout its 120‐year life, by providing a tool for mechanistic understanding and a template for new drug development. DAPT, dual antiplatelet therapy; GI, gastrointestinal; NSAID, nonsteroidal anti‐inflammatory drug; R&D, research and development

Acetylsalicylic acid was marketed as aspirin at the end of the 19th century. Its 120 years of life have been characterized by a few turning points: (i) aspirin started a new drug class twice, first as a nonsteroidal anti‐inflammatory drug (NSAID) and then as an antiplatelet drug^1^, ^2^; (ii) it was cannibalized over time by other members of the new class, largely based on unsubstantiated superiority claims; (iii) it was rescued by the medical/scientific community and repurposed in other therapeutic areas on the basis of newly discovered properties (Figure 1).^3^

In particular, during the past 20 years, we have witnessed the following trends in aspirin usage: (i) a “dropping” trend, characterized by the early discontinuation of low‐dose aspirin from dual antiplatelet therapy (DAPT) or triple antithrombotic therapy (TAT) (ie, oral anticoagulation [OAC])^4^ plus DAPT in patients with atrial fibrillation (AF) following an acute coronary syndrome (ACS) or after percutaneous coronary intervention (PCI); (ii) a “combinatorial” trend, featuring the addition of a lower dose of a P2Y_12_ inhibitor or direct oral anticoagulant (DOAC) to low‐dose aspirin for the long‐term treatment of stable patients with atherosclerotic cardiovascular disease (ASCVD)^5^, ^6^; and (iii) a “repurposing” trend, characterized by growing interest in the oncologic community to assess the chemopreventive effect of aspirin against certain cancers, particularly of the gastrointestinal (GI) tract, as both primary prevention and adjuvant therapy.^7^, ^8^

The aim of this review is to present the mechanistic rationale underlying these trends and discuss the design and main findings of trials testing novel treatment regimens or exploring new therapeutic applications of aspirin.

## 2 DROPPING ASPIRIN FROM DAPT AND TAT: IS LESS MORE?

### 2.1 Rationale for dropping aspirin from DAPT and TAT

To start with the dropping trend, this was nicely articulated by a group of interventional cardiologists in a review article^9^ questioning the role of aspirin in secondary prevention and asking the provocative question: *Is less more?* A major argument supporting their reasoning was a controversial finding suggesting that P2Y_12_ inhibitors may inhibit thromboxane A_2_ (TXA_2_) production to the same extent as aspirin, making the use of aspirin redundant when combined with an effective P2Y_12_ blocker.^10^ This finding was reported in a letter to the editor published in 2010 and was based on a short‐term study in healthy volunteers.^10^ The authors measured the effects of a week’s treatment with low‐dose aspirin or standard‐dose clopidogrel on thromboxane metabolite excretion in 16 healthy volunteers and found a similar 60% reduction in this noninvasive index of platelet activation.^10^ They concluded that “If P2Y_12_ antagonists alone can inhibit platelet TXA_2_ activation pathways, this may explain why addition of aspirin to P2Y_12_ antagonists is not necessarily associated with any improvement in efficacy.”^10^ Accordingly, the “less‐is‐more” concept was proposed in an effort to mitigate the bleeding liability of DAPT while preserving antithrombotic efficacy, achieved through the concomitant inhibition of multiple platelet activation pathways, thereby trying to optimize net clinical benefit.^9^ However, the mechanistic rationale underlying this concept was subsequently questioned by the contradictory findings of Scavone et al,^11^ who reported that inherited deficiency or pharmacologic inhibition of platelet P2Y_12_ does not affect the platelet capacity to synthesize TXA_2_.

### 2.2 Trials dropping aspirin from DAPT

A major test of the “less‐is‐more” concept was provided by the GLOBAL‐LEADERS study.^12^ This was a randomized controlled trial (RCT) of 16 000 patients with either ACS or stable coronary artery disease (CAD) undergoing PCI, in whom this hypothesis was tested to demonstrate superiority of an experimental treatment strategy dropping aspirin after the first month of DAPT, and continuing with ticagrelor monotherapy for the next 23 months, over the reference treatment strategy of DAPT for the first 12 months, followed by aspirin monotherapy for the second year.^12^ The primary end point for superior effectiveness of the experimental strategy was the cumulative 2‐year composite of all‐cause mortality and new Q‐wave myocardial infarction (MI).^12^ At 2 years, there was no statistically significant difference in the rate of the primary end point nor in all‐cause mortality. As one would expect from two equally effective antiplatelet agents, the cumulative incidence of Bleeding Academic Research Consortium 3 or 5 bleeding in the two arms was not significantly different, and their Kaplan‐Meier curves during the second year (when one arm was assigned to receive low‐dose aspirin alone and the other ticagrelor alone) were largely superimposable.^12^

A recent systematic review and meta‐analysis of 5 RCTs (including GLOBAL‐LEADERS) assessing the safety and efficacy of discontinuing aspirin 1 to 3 months after PCI with continued P2Y_12_ inhibitor monotherapy compared with traditional DAPT, involving 32 145 patients with either stable CAD or ACS, found that early discontinuation of aspirin therapy reduced the risk of major bleeding (using heterogeneous outcome definitions as used in each trial) by 40% compared with DAPT (2.0% vs 3.1%; hazard ratio [HR], 0.60; 95% confidence interval [CI], 0.45‐0.79).^13^ There was no apparent increase in the risk of major adverse cardiovascular events (MACEs) (2.7% vs 3.1%; HR, 0.88; 95% CI, 0.77‐1.02).^13^

It is interesting to note that among the recommendations of the 2015 European Society of Cardiology Guidelines for the Management of ACS in Patients Presenting Without Persistent ST‐Segment Elevation (NSTE‐ACS), “P2Y_12_ inhibitor administration for a shorter duration of 3‐6 months after DES [drug‐eluting stent] implantation may be considered in patients deemed at high bleeding risk,” that is, a strategy of dropping clopidogrel or other P2Y_12_ blocker after 3–6 months (instead of 12 months) and continuing with aspirin monotherapy.^14^ This recommendation was based on meta‐analysis of seven RCTs in approximately 16 000 patients that showed a roughly 40% reduction in major bleeding and no significant difference in ischemic events with early discontinuation of P2Y_12_ inhibitor therapy.^14^

Within the limitations of indirect comparisons, the results of these meta‐analyses^13^, ^15^ suggest that following PCI, early discontinuation of low‐dose aspirin has a similar effect on bleeding liability as a shorter duration of P2Y_12_ inhibitor therapy, as would be expected from reduced interference with two equally important mechanisms of platelet activation, that is, TXA_2_ and ADP, respectively.^16^

### 2.3 Trials dropping aspirin from TAT

A second aspect of the “less‐is‐more” paradigm is represented by the withholding of aspirin among patients with chronic coronary syndromes or ACS and AF who require OAC therapy and may undergo PCI with TAT.^17^ A justification for reconsidering the role of aspirin in this setting is related to the fact that “the benefit of a given therapy can change over time, as contemporary treatments develop and disease demographics evolve.”^17^ This obvious statement implies that, under the present circumstances, the control event rate is appreciably lower than in earlier trials, and therefore the absolute benefit of adding aspirin would be diminished as compared to trials performed in the past. However, it does not necessarily follow that there would be a qualitative change in the added benefit of aspirin therapy, as reflected by the relative risk reduction in serious vascular events (SVEs) in any given clinical setting. An example to make this point is represented by A Study of Cardiovascular Events iN Diabetes (ASCEND), a contemporary, United Kingdom–based, placebo‐controlled, primary prevention trial in over 15 000 optimally treated (including statin therapy in 75%) participants with diabetes mellitus.^18^ As compared to placebo, low‐dose aspirin reduced SVEs by 12% (9.6% vs 8.5% in placebo vs aspirin arm, respectively; HR, 0.88; 95% CI, 0.79‐0.97; *P *= .01), a figure remarkably similar to the benefit previously reported by a meta‐analysis of the six largest primary prevention trials in mostly nondiabetic subjects performed in the 1980s and 1990s (HR, 0.88; 95% CI, 0.82–0.94; *P* < .0001).^19^ This estimate was based on roughly 1400 SVEs, giving ASCEND adequate statistical power to detect a moderate treatment effect, as calculated by the Antithrombotic Trialists Collaboration based on 3554 SVEs.^19^

A second line of evidence questioning the validity of the assumption that aspirin may be less effective for secondary prevention. in the context of other contemporary cardiovascular (CV) treatments, than estimated some 30 to 40 years ago, comes from a number of RCTs designed to assess the superiority of other antithrombotic agents in head‐to‐head comparisons versus low‐dose aspirin. If aspirin were indeed less effective than previously estimated, then either newer antiplatelet agents, like ticagrelor, or a DOAC, like rivaroxaban, would be easily shown more effective than aspirin in patients at high CV risk. However, as shown in Table 1, three contemporary RCTs in over 56 000 high‐risk patients, that is, Acute Stroke or Transient Ischemic Attack Treated With Aspirin or Ticagrelor and Patient Outcomes (SOCRATES),^20^ GLOBAL‐LEADERS,^12^ and Cardiovascular Outcomes for People Using Anticoagulation Strategies (COMPASS)^6^ failed to demonstrate superiority of these antithrombotic agents over low‐dose aspirin, consistent with undiminished efficacy of aspirin in diverse and contemporary secondary prevention settings.

**TABLE 1:** Randomized comparisons of other antithrombotic agents versus low‐dose aspirin in high‐risk patients

Trial (n)	Clinical setting	Comparator	HR (95% CI)	*P* value
SOCRATES^20^ (13 199)	Acute stroke or TIA	Ticagrelor	0.89 (0.78–1.01)	.07
GLOBAL LEADERS^a^, ^12^ (15 968)	Post‐PCI	Ticagrelor	0.97 (0.77–1.22)	.79
COMPASS^6^ (27 395)	Stable CVD	Rivaroxaban	0.90 (0.79–1.03)	.12

Abbreviations: CI, confidence interval; CVD, cardiovascular disease; HR, hazard ratio; PCI, percutaneous coronary intervention; TIA, transient ischemic attack.

^a^ Data from the landmark analysis from 1 to 2 years.

Four company‐funded, open‐label RCTs have addressed the question of what is the most appropriate antithrombotic regimen to manage patients with AF experiencing ACS and/or PCI, in view of the competing ischemic and bleeding risks, that is: i) What Is the Optimal Antiplatelet and Anticoagulant Therapy in Patients With Oral Anticoagulation and Coronary Stenting (WOEST)^21^; ii) Open‐Label, Randomized, Controlled, Multicenter Study Exploring Two Treatment Strategies of Rivaroxaban and a Dose‐Adjusted Oral Vitamin K Antagonist Treatment Strategy in Subjects With Atrial Fibrillation Who Undergo Percutaneous Coronary Intervention (PIONEER AF‐PCI)^22^; iii) Randomized Evaluation of Dual Antithrombotic Therapy with Dabigatran Versus Triple Therapy With Warfarin in Patients With Nonvalvular Atrial Fibrillation Undergoing Percutaneous Coronary Intervention (RE‐DUAL PCI)^23^; and iv) the Open‐Label, 2 × 2 Factorial, Randomized, Controlled Clinical Trial to Evaluate the Safety of Apixaban Versus Vitamin K Antagonist and Aspirin Versus Aspirin Placebo in Patients With Atrial Fibrillation and Acute Coronary Syndrome and/or Percutaneous Coronary Intervention (AUGUSTUS).^24^ They ranged in size from 563 to 4614 patients, with a follow‐up ranging from 6 to 14 months. They varied in trial design (two to four treatment arms), patient population, and definition of safety and efficacy outcomes. All four trials used varying bleeding definitions as the primary outcome, but none had major bleeding as the primary end point of the intervention, because of inadequate statistical power (the number of Thrombolysis in Myocardial Infarction [TIMI] major bleedings ranged between 25 and 85, in the smallest and largest trials, respectively).^21^, ^22^, ^23^, ^24^ Furthermore, even the largest of these four trials (ie, AUGUSTUS^24^) was not large enough to detect a moderately increased risk in less common but clinically important ischemic outcomes that would be logically expected to result from aspirin‐free antiplatelet therapy as compared to DAPT. In fact, MI (HR, 0.81; 95% CI, 0.59‐1.12), Academic Research Consortium definite or probable stent thrombosis (HR, 0.52; 95% CI, 0.25‐1.08), urgent revascularization (HR, 0.79; 95% CI, 0.51‐1.21), and death from cardiovascular causes (HR, 0.92; 95% CI, 0.63‐1.33) were all numerically lower in the aspirin versus placebo comparison.^24^ As acknowledged by the AUGUSTUS Investigators, “avoiding aspirin resulted in a 47% lower risk of bleeding than using aspirin and in a nonsignificantly higher incidence of coronary ischemic events. This finding suggests that the price for a significantly lower incidence of bleeding events without aspirin may be a modestly higher risk of coronary ischemic events.”^24^ Given the uncertain prognostic significance of the varying bleeding definitions used in these trials, it is questionable that paying the price of a higher risk of coronary ischemic events would be in the best interest of the patients.

As could be easily predicted from established pathophysiologic and pharmacologic knowledge, these four trials consistently showed reduced bleeding outcomes when comparing OAC plus a P2Y_12_ inhibitor versus OAC plus DAPT.^21^, ^22^, ^23^, ^24^ However, given comparable efficacy and safety of P2Y_12_ inhibitors (both clopidogrel and ticagrelor) and low‐dose aspirin,^25^ the clinically relevant question is not “what happens if I drop aspirin and look at bleeding,” but rather “what is the difference in efficacy and safety between an aspirin‐free versus a P2Y_12_ inhibitor‐free regimen versus DAPT?” Clearly, none of the four RCTs was designed to address this more important question that would have required an additional arm and a much larger sample size. From a mechanistic point of view, the assumption of trials testing the hypothesis that omitting aspirin from the antithrombotic regimen would be associated with less bleeding and no increase in SVEs, implies that platelet TXA_2_ biosynthesis continues to have an important role in primary hemostasis but is no longer relevant to atherothrombosis. The biological plausibility of this assumption seems highly questionable. While a recent meta‐analysis of the four trials concluded that “strategies omitting aspirin caused less bleeding, including intracranial bleeding, without significant difference in MACE, compared with strategies including aspirin,”^26^ this conclusion is biased by the major design flaws outlined above.

## 3 COMBINING ASPIRIN WITH OTHER ANTITHROMBOTIC AGENTS

### 3.1 Rationale for combining aspirin with other antithrombotic agents

In the early trials of aspirin for prevention of death, MI, and stroke in high‐risk patients, aspirin alone (at any dose) achieved 23% proportional SVE reduction versus placebo.^27^ In particular, aspirin approximately halved the risk of SVE in patients with unstable angina in several placebo‐controlled trials carried out in the 1980s and early 1990s.^27^ However, in more recent trials, the rate of SVE in aspirin‐treated patients following ACS was still >10% at 12 months following the acute event.^28^ In aspirin‐treated patients with stable ASCVD (previous MI or stroke), in spite of a proportional SVE reduction of approximately 25% versus placebo, the recurrence rate of SVE is approximately 3% per year.^6^

Since aspirin targets only one mechanism of platelet activation,^29^ combining drugs that target different platelet activation pathways has been hypothesized to produce additive benefit, possibly overcoming the increase in bleeding complications, which is intrinsically associated with an intensified antiplatelet regimen. Moreover, since arterial thrombosis involves primary (platelets, von Willebrand factor) as well as secondary (coagulation cascade) haemostasis,^30^ another tested strategy consists of combining platelet inhibition with low‐dose aspirin together with the inhibition of coagulation factor(s).

### 3.2 With an anticoagulant

Early attempts adding an oral anticoagulant to low‐dose aspirin used warfarin (vitamin K antagonist [VKA]) in patients with MI. The full‐dose warfarin and low‐dose aspirin combination was associated with a reduction in death, nonfatal MI, or thromboembolic stroke, as compared to aspirin alone.^31^, ^32^ However, a significant increase in major bleeding was consistently observed across different studies. Moreover, major drawbacks of warfarin are the need for international normalized ratio monitoring, clinically relevant drug‐drug interactions (DDIs), and low drug adherence.^33^ The development of DOACs, especially those targeting factor Xa (FXa), allowed modulation of the degree of inhibition of a single coagulation factor,^34^ associated with better patient compliance over chronic usage due to lack of monitoring and reduced DDIs. Thus, various degrees of FXa inhibition were tested in association with low‐dose aspirin alone or with DAPT.

The Apixaban for Prevention of Acute Ischemic Events 2 **(**APPRAISE‐2) trial combined a full dose of the anti‐FXa apixaban with aspirin alone or with DAPT following ACS.^35^ The trial was interrupted prematurely for an excess in major bleeding (2.7% vs 1.1% in apixaban vs placebo, respectively; HR, 2.59; 95% CI, 1.50–4.46; *P *= .001), without any detectable benefit. The phase II Anti‐Xa Therapy to Lower Cardiovascular Events in Addition to Standard Therapy in Subjects with Acute Coronary Syndrome–Thrombolysis in Myocardial Infarction 46 (ATLAS‐ACS TIMI 46) study showed a dose‐dependent increase in bleeding requiring medical attention for rivaroxaban doses between 5 and 20 mg, either given as a single or divided daily doses in patients following ACS.^36^ This clinical outcome paralleled the increasing degree of anti‐FXa activity measured across the same dose range.^34^, ^37^ Based on these findings, the ATLAS‐ACS TIMI 51 trial tested the addition of the lowest doses (2.5 and 5 mg twice daily) of rivaroxaban to DAPT with aspirin and clopidogrel in patients following ACS.^38^ The 2.5‐mg twice‐daily dose was associated with a significant SVE reduction versus placebo (9.1% vs 10.7%, respectively; HR, 0.84; 95% CI, 0.72‐0.97; *P* = .008; number needed to treat [NNT] = 63) with a significant increase in TIMI major bleeding (1.8% vs 0.6%; HR, 3.46; 95% CI, 2.08‐5.77; *P* < .001; number needed to harm [NNH] = 83).^38^ There was also a significant increase in major bleeding with the 5‐mg twice‐daily dose versus 2.5 mg twice daily.

The COMPASS trial tested the combination of low‐dose rivaroxaban (2.5 mg twice daily) with low‐dose aspirin in stable patients with ASCVD (after MI or with symptomatic peripheral artery disease [PAD]).^6^ SVE occurred in 4.1% versus 5.4% in the aspirin/rivaroxaban versus aspirin arms (HR, 0.76; 95% CI, 0.66‐0.86; *P* < .001; NNT = 77), respectively, with a reduction in CV mortality (1.7% vs 2.2%, respectively; HR, 0.82; 95% CI, 0.71‐0.96; *P* = .01). Major bleeding was significantly increased in the combination arm (3.1% vs 1.9%; HR, 1.70; 95% CI, 1.40‐2.05; *P* < .001; NNH = 83) (Table 2), without significant differences in fatal bleeding, intracranial hemorrhage, or critical organ bleeding.^6^ Based on the encouraging results in the subgroup of patients with symptomatic PAD, the Vascular Outcomes Study of ASA Along With Rivaroxaban in Endovascular or Surgical Limb Revascularization for PAD (VOYAGER PAD) tested the combination aspirin/low‐dose rivaroxaban against aspirin alone in patients with symptomatic PAD enrolled within 10 days from revascularization.^39^ The primary composite outcome of acute limb ischemia, major amputation for vascular causes, MI, ischemic stroke, or CV death at 3 years occurred in 15.5% and 17.8% of the rivaroxaban and placebo groups (HR, 0.85; 95% CI, 0.76‐0.96; *P* = .009; NNT = 44), respectively, while ISTH‐defined major bleeding occurred in 5.9% versus 4.1%, respectively (HR, 1.42; 95% CI, 1.10‐1.84; *P* = .007; NNH = 53).

**TABLE 2:** Benefit/risk ratio in recent randomized controlled trials of antithrombotic therapy for primary and secondary prevention

Trial	NNT	NNH	NNH/NNT
ASCEND^18^	91	112	1.2
COMPASS^6^	77	83	1.1
PEGASUS^5^	79	81	1.0
THEMIS^49^	125	83	0.7

Abbreviations: NNH, number needed to harm; NNT, number needed to treat.

The combination of aspirin with a higher dose of rivaroxaban (10 mg daily) was tested in elderly patients (median age, 80 years) undergoing transcatheter aortic valve replacement on a background of DAPT for the first 3 months following transcatheter aortic valve replacement, followed by aspirin/placebo or aspirin/rivaroxaban,^40^ with the aim of reducing strokes (mostly cardioembolic) associated with valve replacement. This trial was interrupted prematurely for an excess of mortality (mostly non‐CV) and major bleeding in the rivaroxaban group.

Thus, full inhibition of one (FXa) or more (with VKA) coagulation factors combined with aspirin alone or DAPT was associated with major safety concerns. Modulating the inhibition of FXa to lower degrees produced an additional 15% to 24% SVE reduction with an acceptable increase in bleeding complications. It seems likely that, in aspirin‐treated patients, cyclooxygenase (COX)‐1 inhibition may downsize the platelet surface available for coagulation factor assembly, thereby reducing the level of FXa inhibition required for effective antithrombotic activity.

### 3.3 With a P2Y_12_ inhibitor

**FIGURE 2:**
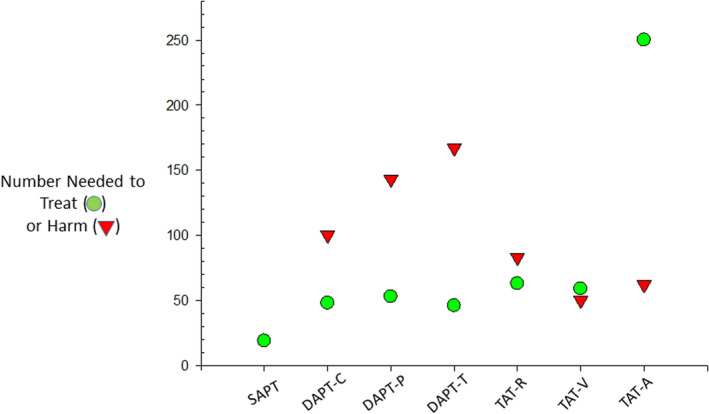
Number needed to treat (NNT) and number needed to harm (NNH) in trials adding one or two antithrombotic drugs to low‐dose aspirin. The figure depicts the NNT and NNH values for 12 months of therapy in patients with acute coronary syndromes treated with aspirin alone (SAPT), aspirin plus clopidogrel (DAPT‐C), aspirin plus prasugrel (DAPT‐P), aspirin plus ticagrelor (DAPT‐T), aspirin plus clopidogrel and low‐dose rivaroxaban (TAT‐R), aspirin plus clopidogrel and vorapaxar (TAT‐V); aspirin plus clopidogrel and full‐dose apixaban (TAT‐A)

The combination of ADP receptor (P2Y_12_) blockade by clopidogrel, prasugrel, or ticagrelor, with low‐dose aspirin has been successfully tested in clinical settings considered suitable for intensifying platelet inhibition, that is, in patients following ACS with or without coronary revascularization.^28^, ^41^, ^42^ The inhibition of a third platelet‐activating pathway by the thrombin receptor protease‐activated receptor‐1 antagonist, vorapaxar, in the same clinical setting was associated with a trend toward a reduction in the primary efficacy end point (15.9% vs. 17.0% in vorapaxar vs placebo, respectively; HR, 0.92; 95% CI, 0.85‐1.01; *P* = .07), with a major bleeding excess offsetting the potential benefit (2.2% vs 1.4% in vorapaxar vs placebo, respectively; HR, 1.35; 95% CI, 1.16‐1.58; *P* < .001),^43^ suggesting the achievement of a ceiling benefit/harm balance with DAPT (Figure 2).

In patients with acute mild to moderate, noncardioembolic stroke or transient ischemic attack, early P2Y_12_ blockade by clopidogrel^44^, ^45^ or ticagrelor^46^ in addition to aspirin has been consistently associated with a significant reduction of ischemic stroke recurrence within the first 3 months in three different trials, with a favorable benefit/harm balance (NNH/NNT ratio of 3.0 for clopidogrel^45^ and 2.7 for ticagrelor^46^). A fourth trial compared the inhibition of three versus two platelet‐activating pathways by adding dipyridamole, a cyclic AMP‐phosphodiesterase inhibitor, to aspirin and clopidogrel.^47^ Similarly to the disappointing results obtained with triple antiplatelet therapy in ACS,^43^ the inhibition of three platelet pathways in patients with mild to moderate stroke showed no further benefit but a substantially increased bleeding risk that caused the early termination of the trial.^47^

The Prevention of Cardiovascular Events in Patients with Prior Heart Attack Using Ticagrelor Compared to Placebo on a Background of Aspirin (PEGASUS) TIMI 54 trial tested the addition of two different doses (60 and 90 mg twice daily) of ticagrelor on top of low‐dose aspirin, in stable patients with a MI in the previous 1 to 3 years and at least one additional CV risk factor.^5^ The lower dose of ticagrelor reduced the 3‐year SVE rate by 16%, from 9.04% in the placebo group to 7.77% (HR, 0.84; 95% CI, 0.74‐0.95; *P* = .004; NNT = 79) with no statistically significant reduction in mortality.^5^ Rates of TIMI major bleeding were significantly higher with ticagrelor (2.30% with 60 mg) than with placebo (1.06%) (HR, 2.32; 95% CI, 1.68‐3.21; *P* < .001; NNH = 81) (Table 2), but the rates of fatal bleeding or nonfatal intracranial hemorrhage did not differ significantly between either ticagrelor dose and placebo.^5^

Patients with diabetes and stable CAD are also considered at higher risk among the diabetes population without a prior SVE.^48^ In the Effect of Ticagrelor on Health Outcomes in Diabetes Mellitus Patients Intervention Study (THEMIS), intensifying the antiplatelet regimen by adding ticagrelor (60 mg twice daily) to low‐dose aspirin in this clinical setting was associated with a modest SVE reduction (from 8.5% to 7.7%; HR, 0.90; 95% CI, 0.81‐0.99; *P* = .04), and a disproportionate increase in TIMI major bleeding (from 1% to 2.2%; HR, 2.32; 95% CI, 1.82‐2.94; *P* < .001) resulting in a NNH/NNT ratio <1 (Table 2).^49^

While combining platelet COX‐1 inhibition with low‐intensity FXa inhibition appears to achieve a proportionally larger benefit in preventing SVE in stable patients with ASCVD than the combination of platelet COX‐1 and P2Y_12_ inhibition, with a comparable increase in bleeding risk, the actual superiority of one approach versus the other would require a very large randomized comparison between the two in order to be reliably assessed.

## 4 REPURPOSING ASPIRIN

### 4.1 Rationale for repurposing aspirin

**FIGURE 3:**
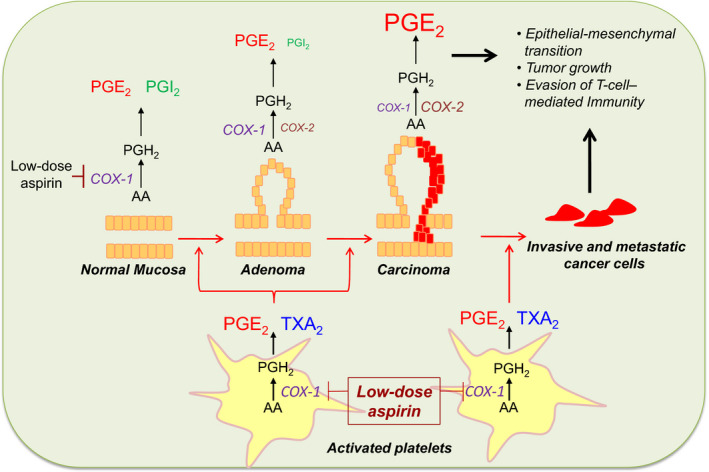
The platelet contribution to colorectal cancer development: early versus late phases. In the first stages of intestinal tumorigenesis, platelets may play a key role, since they are activated in response to mucosal injury, and release various soluble mediators, for example, thromboxane A_2_ (TXA_2_), prostaglandin E_2_ (PGE_2_), and various growth factors, that may contribute to the induction of several signaling pathways associated with a phenotypic switch of the stromal cells. In this scenario, the activation of stromal cells, in turn, can lead to abnormal expression of cyclooxygenase (COX)‐2 in epithelial cells. These molecular events lead to enhanced biosynthesis of the pro‐tumorigenic prostanoid, PGE_2_, which is generated mainly by COX‐1 in the normal mucosa and by COX‐1/COX‐2 and COX‐2 in the adenoma and adenocarcinoma, respectively. In addition, in the late phase of tumorigenesis, tumor cells may enter into the circulation and interact with platelets. The adhesion of platelets to tumor cells leads to platelet activation and their release of mediators that induce phenotypic changes in tumor cells thereby facilitating their extravasation and colonization of distal organs. The anticancer effect of low‐dose aspirin may also be explained by the fact that the drug, in addition to platelet COX‐1, is able to acetylate and inhibit COX‐1 in intestinal epithelial cells, thus preventing both the early and late phases of intestinal tumorigenesis. Reproduced from Patrignani and Patrono,^8^ with permission

Another trend opening the prospect of a third life of aspirin is represented by its repurposing in other areas of medicine. A clear new avenue is represented by its potential role in oncology, primarily for the prevention of colorectal cancer (CRC) and perhaps other tumors.^50^ During the past 20 years, a large body of observational and randomized evidence has accumulated consistently suggesting that aspirin can reduce the risk of recurrence of a sporadic colorectal adenoma, prevent the development of CRC, and reduce associated metastases.^7^ Given that these clinical findings were reported in association with the use of an antiplatelet regimen of low‐dose aspirin, they have been interpreted as reflecting an important role of local platelet activation at different stages of colorectal carcinogenesis, as supported by experimental studies in murine models of the disease and depicted in Figure 3.^3^, ^8^

Moreover, the role of aspirin in the treatment of osteoarthritis (OA) and rheumatoid arthritis (RA), largely dwarfed by the advent of a large family of NSAIDs,^2^ should be reassessed in light of new scientific evidence and regulatory constraints on the use of NSAIDs, whereby aspirin is the only NSAID without a Food and Drug Administration black‐box warning on untoward CV effects (Table 3).

**TABLE 3:** Safety and regulatory issues with the use of aspirin, traditional nonsteroidal anti‐inflammatory drugs, and coxibs in the treatment of osteoarthritis

Variable	Aspirin	tNSAIDs	Coxibs
RR of serious vascular events	0.81–0.88^a^	1.4^b^	1.4^b^
Interference with the antiplatelet effect of aspirin	No	Yes	No
RR of upper GI complications	4.0–7.6^c^	1.9–4.2^b^	1.8^b^
Contraindication for use in patients at high CV risk	No	Yes	Yes

Abbreviations: CV, cardiovascular; GI, gastrointestinal; RR, relative risk; tNSAIDs, traditional nonsteroidal anti‐inflammatory drugs.

^a^ Antithrombotic Trialists Collaboration, Lancet 2009.^19^

^b^ Coxib Traditional NSAIDA Trialists Collaboration, Lancet 2013.^68^

^c^ García Rodríguez, 1998.^67^

### 4.2 In cancer prevention and treatment

The observation of lower rates of CRC in populations exposed to regular use of aspirin and other NSAIDs triggered interest in assessing whether long‐term administration of these drugs may be chemopreventive against CRC and other cancers.^7^, ^8^, ^50^, ^51^ The race began some 20 years ago, with simultaneous initiation of placebo‐controlled RCTs of coxibs, a new class of selective COX‐2 inhibitors,^52^ and aspirin (mostly low‐dose) in subjects with sporadic colorectal adenomas for the prevention of adenoma recurrence. A highly selective coxib (eg, rofecoxib) offered the prospect of improved GI safety over traditional NSAIDs for long‐term treatment. Unfortunately, this promising line of research was abruptly discontinued because of the emergence of a COX‐2–dependent CV hazard of coxibs (and other NSAIDs).^53^ However, the interest in using aspirin as a pharmacologic tool to probe the chemoprevention hypothesis was reinforced by the results of several placebo‐controlled aspirin RCTs demonstrating a comparable effect in reducing colorectal adenoma recurrence as reported by coxib trials in the same setting.^7^ These early promising findings prompted additional multidisciplinary studies with largely different methodologies. Based on their results, several lines of evidence strongly suggest an association between aspirin treatment and reduced risk of GI cancers, with just a few establishing causality between the two.^7^, ^8^ These heterogeneous but remarkably consistent pieces of evidence include (i) >50 observational case‐control studies and a meta‐analysis confirming the original observation reported by Kune et al, with a meta‐analytic estimate of 38% lower risk of CRC associated with regular use of aspirin^51^, ^54^; (ii) four placebo‐controlled aspirin RCTs in subjects with sporadic colorectal adenomas, and their meta‐analysis demonstrating a 20% to 30% reduced risk of recurrence^55^; (iii) a placebo‐controlled aspirin RCT in patients with Lynch syndrome demonstrating 35% lower risk of developing CRC during 10‐year follow‐up (HR, 0.65; 95% CI, 0.43‐0.97; *P* = .035)^56^, ^57^; (iv) long‐term (up to 20 years), prospective follow‐up of the Women’s Health Study, a placebo‐controlled RCT of aspirin 100 mg every other day for primary CV prevention, showing a 20% lower risk of CRC^58^; (v) a post hoc, individual patient data (IPD) meta‐analysis of 51 RCTs with ≈77 000 participants recruited into aspirin trials for both primary and secondary prevention of vascular events, suggesting short‐term reductions in cancer incidence and mortality^59^; (vi) a post hoc IPD meta‐analysis of seven aspirin CV prevention RCTs with mean duration of scheduled trial treatment of 4 years or longer in 23 535 patients, suggesting that benefit in reducing cancer mortality was apparent only after 5 years’ follow‐up and increased with scheduled duration of trial treatment^60^; and (vii) a post hoc analysis of five large RCTs of daily aspirin (≥75 mg daily) versus control for the prevention of vascular events in the United Kingdom (17 285 trial participants) suggesting that allocation to aspirin reduced risk of cancer with distant metastasis.^61^

Different explanations have been put forward to explain the apparent chemopreventive effect of aspirin.^7^, ^8^, ^61^ These include mechanisms related to its anti‐inflammatory effect, platelet inhibition at sites of GI mucosal injury, and others. Uncertainty concerning aspirin’s mechanism of action in protecting against CRC has resulted in a wide range of daily doses being used in newly designed trials to test its chemopreventive effect, from as low as 81 mg used in one of the adenoma recurrence prevention trials^55^ to as high as 600 mg used in the the Colorectal Adenoma/Carcinoma Prevention Programme (CAPP)‐2 trial of patients with Lynch syndrome.^56^ Similarly, aspirin doses used in the CV prevention trials ranged between 75 mg and 1200 mg daily.^59^, ^60^, ^62^ Interestingly, the analyses of both chemopreventive and CV RCTs did not disclose any apparent dose dependence of the protective effect of aspirin against adenoma recurrence or CRC prevention. Consistently with similar results of observational studies, these findings clearly indicate saturability of the chemopreventive effect of aspirin at low doses taken once daily, that is, the same requirements for its antiplatelet effect.^1^, ^3^

This very large body of evidence has resulted in at least three important consequences during the past 5 years. First, in 2016, the United States Preventive Services Task Force (USPSTF), an independent organization funded by the US Department of Health, issued a grade B recommendation for the use of low‐dose aspirin (75‐100 mg/day) “for the primary prevention of CVD and CRC in adults 50‐59 years of age who have a 10% or greater 10‐year CVD risk, are not at increased risk for bleeding, have a life expectancy of at least 10 years, and are willing to take low‐dose aspirin daily for at least 10 years.”^63^ Furthermore, the USPSTF recommended that “the decision to initiate low‐dose aspirin use for the primary prevention of CVD and CRC in adults age 60‐69 years who have a 10% or greater 10‐year CVD risk should be an individual one” (grade C).^63^ The USPSTF found no adequate evidence to recommend in favor or against the use of aspirin in people aged ≥70.^63^ When considering the wording of the USPSTF recommendation, it should be emphasized that it does not represent an endorsement of low‐dose aspirin for the chemoprevention of CRC, but rather an acknowledgment that lowering the long‐term risk of CRC may represent an additional benefit of antiplatelet prophylaxis for primary CV prevention.^7^

Second, the evidence supporting a chemopreventive effect of aspirin provided the rationale and motivation for the design of new RCTs testing the efficacy and safety of aspirin in the adjuvant setting. A number of RCTs evaluating aspirin use after primary radical therapy are ongoing. The Add‐Aspirin study includes four phase‐3 RCTs evaluating the effect of daily aspirin on recurrence and survival after radical cancer therapy in four tumor cohorts: gastroesophageal, colorectal, breast, and prostate cancer, with each cohort planning to recruit approximately 2500 patients.^64^ An open‐label run‐in phase (aspirin 100 mg daily for 8 weeks) precedes double‐blind randomization (for participants aged <75 years, aspirin 300 mg, aspirin 100 mg, or matched placebo in a 1:1:1 ratio; for patients aged ≥75 years, aspirin 100 mg or matched placebo in a 2:1 ratio).^64^ A preplanned analysis of feasibility, including recruitment rate, adherence, and toxicity, was recently performed.^64^ This analysis found that aspirin is well tolerated after radical cancer therapy. Toxicity has been low and there was no evidence of a difference in adherence, acceptance of randomization, or toxicity between the different cancer cohorts.^64^ Recruitment is ongoing, with over 7000 patients already randomized and study completion of the different cohorts expected between 2025 and 2027 (R. Langley, personal communication).

Third, on January 29, 2020, the UK National Institute for Health and Care Excellence (NICE) published an update of its CRC Guideline (NG151), which under the heading “Prevention of Colorectal Cancer in People With Lynch syndrome” contains the following recommendation: “Consider daily aspirin, to be taken for more than 2 years, to prevent colorectal cancer in people with Lynch syndrome” (available at nice.org.uk). Although the CAPP‐2 study used aspirin at a daily dose of 600 mg, the NICE CRC Guideline indicates that “commonly used aspirin doses in current practice are 150 mg or 300 mg.” The CAPP‐3 trial will focus on finding the right dose of aspirin for people with a mismatch repair gene defect, the underlying cause of Lynch syndrome. Three thousand people who have Lynch syndrome will be invited to take part in a dose noninferiority trial testing 100 mg, 300 mg, or 600 mg of aspirin per day (further details are available at capp3.org). Furthermore, the Effect of Chemoprevention by Low‐Dose Aspirin on New or Recurrent Colorectal Adenomas in Patients With Lynch Syndrome (AAS‐Lynch) trial, a prospective, multicenter, double‐blind, placebo‐controlled RCT, is currently investigating whether daily aspirin therapy, at a dose of 100 or 300 mg, may decrease the occurrence or recurrence of colorectal adenomas in 852 patients aged <75 years with Lynch syndrome, compared with placebo.^65^

### 4.3 In treating inflammation

Historically, aspirin was the leading NSAID for the first 70 years of the 20th century. Then, the introduction of newer NSAIDs in the 1960s (indomethacin) and 1970s (ibuprofen and naproxen) led to a progressive displacement of aspirin from the NSAID market.^2^ Although synthesized and developed to reproduce the spectrum of aspirin’s pharmacologic activities, ibuprofen and then naproxen were easier to use (no need for monitoring serum salicylate), required fewer tablets per day and less frequent dosing, and displayed an improved GI safety profile based on endoscopic studies.^2^ These findings, together with asymmetrical marketing pressures behind novel NSAIDs versus aspirin, led to a progressive decline of high‐dose aspirin as a first‐line therapeutic option for patients with OA/RA in the 1980s. This decline coincided with the rising interest in low‐dose aspirin as an antiplatelet agent,^1^ culminating in the publication of Second International Study of Infarct Survival (ISIS‐2) in 1988, that characterized low‐dose aspirin as a lifesaving drug in the setting of a suspected acute MI.^66^ Today, aspirin as an analgesic/anti‐inflammatory agent might have a role in patients at high CV risk (including RA patients), for whom other NSAIDs are largely contraindicated or discouraged, because of their proven cardiac toxicity or pharmacodynamic interaction with low‐dose aspirin (Table 3).^67^, ^68^

## 5 ASPIRIN IN THE 2020 ISTH CONGRESS REPORT: FUTURE CHALLENGES

Over 100 abstracts presented at the ISTH 2020 Congress dealt with aspirin either in the title or in the text, in a context of future ways of “repurposing” and “recombining” rather than “retiring.”

Within the repurposing setting, aspirin appears to be the reference antithrombotic drug in the primary CV prevention of rare acquired or inborn diseases of red blood cells, such as polycythemia vera^69^ and thalassemia,^70^ respectively. Even in young people with thalassemia, aspirin may protect their brain from any type of ischemia (arterial, embolic, micro‐ and macrovascular, silent and overt).^71^ The still‐ongoing coronavirus disease 2019 (COVID‐19) pandemics has renewed the attention on the well‐known liaison between infection and haemostasis. Consistently, platelet and endothelial activation documented in patients with COVID‐19^72^ reinforces the rationale for two ongoing trials testing the efficacy of aspirin in mitigating the prothrombotic state, reducing hospitalization and fatal complications of this disease.^73^, ^74^ Aspirin, and possibly other antiplatelet drugs, may face relevant pharmacologic challenges in the near future, which should be addressed to maintain an optimal antithrombotic protection. While in the early trials of aspirin (for either primary or secondary prevention) body size was within the normal‐low weight range,^19^ obesity will increasingly characterize the majority of patients with CV disease in the near future. Obesity by itself is a condition that may affect aspirin pharmacology,^75^ possibly through higher degradation rate by plasma esterases.^76^ Moreover, increased platelet turnover has been associated with a reduced responsiveness to standard once‐daily low‐dose aspirin in primary^77^ or transiently acquired^78^ platelet disorders. A high platelet regeneration rate seems also to modify the pharmacodynamics of the P2Y_12_ inhibitor clopidogrel in patients on DAPT.^79^ Interestingly, *BRAFV600E* mutated mice develop hepatocarcinogenesis similarly to humans with the same mutation, with an associated increase in thrombopoietin, megakaryopoiesis, and platelet deposition in hepatic sinusoids.^80^ These mice were protected from hepatic carcinogenesis by low‐dose aspirin in association with a parallel reduction of platelet deposition in liver sinusoids.^80^

Within the recombining setting, Wong et al^81^ showed that aspirin combined with a novel factor XIa (FXIa) inhibitor did not increase bleeding time in animal models. Since FXIa inhibitors are in phase II/III clinical trials for venous thromboembolism, the association with aspirin may also be of relevance to ASCVD.

## 6 CONCLUSIONS

Despite being 120 years old, aspirin continues to be considered a cornerstone of antithrombotic therapy in ASCVD. It is, in fact, the only antiplatelet agent with current recommendations for its use throughout the CV risk continuum, from primary prevention in people with high‐risk diabetes mellitus to treatment of ACS (Table 4).^48^, ^82^, ^83^

**TABLE 4:** The European Society of Cardiology recommendations on the use of aspirin for cardiovascular disease prevention throughout the cardiovascular risk continuum

Variable	Diabetes mellitus at high/very high risk	Definitive evidence of CAD on imaging	Symptomatic CHD	Prior MI	NSTE‐ACS
Annual rate of SVEs	≈2.0–3.0%	?	≈2.0–4.0%	≈4.0–8.0%	≈10%
Pivotal trial	ASCEND	NA	SAPAT	ATT meta‐analysis	RISC, ATT Meta‐analysis
Strength of ESC recommendation	IIb/A^a^	IIb/C^b^	I/B^b^	I/A^b^	I/A^c^

Abbreviations: ASCEND, A Study of Cardiovascular Events in Diabetes; ATT, Antithrombotic Trialist's Collaboration; CAD, coronary artery disease; CHD, coronary heart disease; ESC, European Society of Cardiology; MI, myocardial infarction; NA, not available; NSTE‐ACS, acute coronary syndromes without persistent ST‐segment elevation; RISC, Research Group on Instability in Coronary Artery Disease; SAPAT, Swedish Angina Pectoris Aspirin Trial; SVEs, serious vascular events.

^a^ 2019 ESC/EASD Diabetes Guidelines.^48^

^b^ 2019 ESC Chronic Coronary Syndromes Guidelines.^83^

^c^ 2020 ESC Acute Coronary Syndromes Guidelines.^82^

We have critically reviewed a current trend for considering aspirin‐free antithrombotic regimens in patients undergoing PCI, and conclude that there is no valid mechanistic rationale nor convincing evidence from RCTs for withdrawing low‐dose aspirin rather than a P2Y_12_ inhibitor from DAPT or TAT in this setting. Under clinical circumstances in which DAPT (or TAT) is the standard of care, studies comparing an aspirin‐free regimen to DAPT are as flawed as those comparing clopidogrel‐free regiment to DAPT, and their results cannot provide unbiased guidance to clinical practice.^84^, ^85^ Furthermore, the results of the GLOBAL‐LEADERS trial do not support the “less‐is‐more” paradigm.^9^

The combined use of low‐dose aspirin with a low‐dose FXa inhibitor^6^ or with an effective P2Y_12_ inhibitor represents a rational approach to reduce residual CV risk of patients with ASCVD, in light of the multifactorial nature of atherothrombosis.^16^ However, we should be aware of the exponential rise in NNT values to obtain an additional reduction in SVE, in view of the relatively low annual event rate and moderate effect size of these interventions in stable patients.

The evidence supporting a chemopreventive effect of low‐dose aspirin against CRC^3^, ^7^, ^8^ was considered sufficiently strong to convince the USPSTF to issue a new recommendation for its use in primary CVD prevention with the additional long‐term benefit of CRC prevention^63^ and to stimulate several oncology networks to initiate new aspirin trials in the adjuvant setting.^64^

Finally, we believe that the role of aspirin in the treatment of OA should be reassessed in light of new scientific evidence and regulatory constraints on the use of currently available NSAIDs.^67^, ^68^

## AUTHOR CONTRIBUTIONS

CP and BR reviewed the literature and wrote the manuscript.

## RELATIONSHIP DISCLOSURE

BR has received consultant and speaker fees from Bayer AG, Novartis, and MedScape. CP reports receiving consultant and speaker fees from Acticor Biotech, Amgen, Bayer, GlaxoSmithKline, Eli Lilly, Tremeau, and Zambon; he chairs the Scientific Advisory Board of the International Aspirin Foundation.

## References

[1] Patrono C . Aspirin as an antiplatelet drug. N Engl J Med. 1994;330:1287‐1294. 10.1056/NEJM199405053301808 8145785

[2] Patrono C , Rocca B . Nonsteroidal antiinflammatory drugs: past, present and future. Pharmacol Res. 2009;59:285‐289. 10.1016/j.phrs.2009.01.011 19416627

[3] Patrono C . The multifaceted clinical readouts of platelet inhibition by low‐dose aspirin. J Am Coll Cardiol. 2015;66:74‐85. 10.1016/j.jacc.2015.05.012 26139061

[4] Capodanno D , Huber K , Mehran R , et al. Management of antithrombotic therapy in atrial fibrillation patients undergoing PCI: JACC state‐of‐the‐art review. J Am Coll Cardiol. 2019;74:83‐99. 10.1016/j.jacc.2019.05.016 31272556

[5] Bonaca MP , Bhatt DL , Cohen M , et al. PEGASUS‐TIMI 54 steering committee and investigators. Long‐term use of ticagrelor in patients with prior myocardial infarction. N Engl J Med. 2015;372:1791‐1800. 10.1056/NEJMoa1500857 25773268

[6] Eikelboom JW , Connolly SJ , Bosch J , et al. Rivaroxaban with or without aspirin in stable cardiovascular disease. N Engl J Med. 2017;377:1319‐1330. 10.1056/NEJMoa1709118 28844192

[7] Patrignani P , Patrono C . Aspirin and cancer. J Am Coll Cardiol. 2016;68:967‐976. 10.1016/j.jacc.2016.05.083 27561771

[8] Patrignani P , Patrono C . Aspirin, platelet inhibition and cancer prevention. Platelets. 2018;29:779‐785. 10.1080/09537104.2018.1492105 29985750

[9] Gargiulo G , Windecker S , Vranckx P , Gibson CM , Mehran R , Valgimigli M . A critical appraisal of aspirin in secondary prevention: is less more? Circulation. 2016;134:1881‐1906. 10.1161/CIRCULATIONAHA.116.023952 27920074

[10] Armstrong PC , Dhanji AR , Tucker AT , Mitchell JA , Warner TD . Reduction of platelet thromboxane A2 production ex vivo and in vivo by clopidogrel therapy. J Thromb Haemost. 2010;8:613‐615. 10.1111/j.1538-7836.2009.03714.x 19995405

[11] Scavone M , Femia EA , Caroppo V , Cattaneo M . Inhibition of the platelet P2Y12 receptor for adenosine diphosphate does not impair the capacity of platelet to synthesize thromboxane A2. Eur Heart J. 2016;37:3347‐3356. 10.1093/eurheartj/ehv551 26516174

[12] Vranckx P , Valgimigli M , Juni P , et al. Ticagrelor plus aspirin for 1 month, followed by ticagrelor monotherapy for 23 months vs aspirin plus clopidogrel or ticagrelor for 12 months, followed by aspirin monotherapy for 12 months after implantation of a drug‐eluting stent: a multicentre, open‐label, randomised superiority trial. Lancet. 2018;392:940‐949. 10.1016/S0140-6736(18)31858-0 30166073

[13] O’Donoghue ML , Murphy SA , Sabatine MS . The safety and efficacy of aspirin discontinuation on a background of a P2Y12 inhibitor in patients after percutaneous coronary intervention: a systematic review and meta‐analysis. Circulation. 2020;142:538‐545. 10.1161/CIRCULATIONAHA.120.046251 32551860

[14] Roffi M , Patrono C , Collet JP , et al. ESC guidelines for the management of acute coronary syndromes in patients presenting without persistent ST‐segment elevation: task force for the management of acute coronary syndromes in patients presenting without persistent ST‐segment elevation of the European Society of Cardiology (ESC). Eur Heart J. 2015;2016(37):267‐315. 10.1093/eurheartj/ehv320 26320110

[15] Navarese EP , Andreotti F , Schulze V , et al. Optimal duration of dual antiplatelet therapy after percutaneous coronary intervention with drug eluting stents: meta‐analysis of randomised controlled trials. BMJ. 2015;350:h1618. 10.1136/bmj.h1618 25883067 PMC4410620

[16] Davi G , Patrono C . Platelet activation and atherothrombosis. N Engl J Med. 2007;357:2482‐2494. 10.1056/NEJMra071014 18077812

[17] Jacobsen AP , Raber I , McCarthy CP , et al. Lifelong aspirin for all in the secondary prevention of chronic coronary syndrome: still sacrosanct or is reappraisal warranted? Circulation. 2020;142:1579‐1590. 10.1161/CIRCULATIONAHA.120.045695 32886529

[18] Bowman L , Mafham M , Wallendszus K , et al. Effects of aspirin for primary prevention in persons with diabetes mellitus. N Engl J Med. 2018;379:1529‐1539. 10.1056/NEJMoa1804988 30146931

[19] Collaboration Antithrombotic Trialists , Baigent C , Blackwell L , et al. Aspirin in the primary and secondary prevention of vascular disease: collaborative meta‐analysis of individual participant data from randomised trials. Lancet. 2009;373:1849‐1860. 10.1016/S0140-6736(09)60503-1 19482214 PMC2715005

[20] Johnston SC , Amarenco P , Albers GW , et al. Ticagrelor versus aspirin in acute stroke or transient ischemic attack. N Engl J Med. 2016;375:35‐43. 10.1056/NEJMoa1603060 27160892

[21] Dewilde WJ , Oirbans T , Verheugt FW et al. Use of clopidogrel with or without aspirin in patients taking oral anticoagulant therapy and undergoing percutaneous coronary intervention: an open‐label, randomised, controlled trial. The Lancet. 2013;381(9872):1107‐1115. 10.1016/S0140-6736(12)62177-1 23415013

[22] Gibson CM , Mehran R , Bode C , et al. Prevention of bleeding in patients with atrial fibrillation undergoing PCI. N Engl J Med. 2016;375:2423‐2434. 10.1056/NEJMoa1611594 27959713

[23] Cannon CP , Bhatt DL , Oldgren J , et al. RE‐DUAL PCI steering committee investigators. Dual antithrombotic therapy with dabigatran after PCI in atrial fibrillation. N Engl J Med. 2017;377:1513‐1524. 10.1056/NEJMoa1708454 28844193

[24] Lopes RD , Heizer G , Aronson R , et al. Antithrombotic therapy after acute coronary syndrome or PCI in atrial fibrillation. N Engl J Med. 2019;380:1509‐1524. 10.1056/NEJMoa1817083 30883055

[25] Chiarito M , Sanz‐Sanchez J , Cannata F , et al. Monotherapy with a P2Y12 inhibitor or aspirin for secondary prevention in patients with established atherosclerosis: a systematic review and meta‐analysis. Lancet. 2020;395:1487‐1495. 10.1016/S0140-6736(20)30315-9 32386592

[26] Lopes RD , Hong H , Harskamp RE , et al. Safety and efficacy of antithrombotic strategies in patients with atrial fibrillation undergoing percutaneous coronary intervention: a network meta‐analysis of randomized controlled trials. JAMA Cardiol. 2019;4:747‐755. 10.1001/jamacardio.2019.1880 31215979 PMC6584885

[27] Antithrombotic Trialists Collaboration . Collaborative meta‐analysis of randomised trials of antiplatelet therapy for prevention of death, myocardial infarction, and stroke in high risk patients. BMJ. 2002;324:71‐86. 10.1136/bmj.324.7329.71 11786451 PMC64503

[28] Yusuf S , Zhao F , Mehta SR , Chrolavicius S , Tognoni G , Fox KK . Clopidogrel in unstable angina to prevent recurrent events trial I. Effects of clopidogrel in addition to aspirin in patients with acute coronary syndromes without ST‐segment elevation. N Engl J Med. 2001;345:494‐502. 10.1056/NEJMoa010746 11519503

[29] Patrono C , Garcia Rodriguez LA , Landolfi R , Baigent C . Low‐dose aspirin for the prevention of atherothrombosis. N Engl J Med. 2005;353:2373‐2383. 10.1056/NEJMra052717 16319386

[30] Hoshiba Y , Hatakeyama K , Tanabe T , Asada Y , Goto S . Co‐localization of von Willebrand factor with platelet thrombi, tissue factor and platelets with fibrin, and consistent presence of inflammatory cells in coronary thrombi obtained by an aspiration device from patients with acute myocardial infarction. J Thromb Haemost. 2006;4:114‐120. 10.1111/j.1538-7836.2005.01701.x 16409460

[31] van Es RF , Jonker JJ , Verheugt FW , Deckers JW , Grobbee DE . Antithrombotics in the Secondary Prevention of Events in Coronary Thrombosis‐2 Research Group. Aspirin and coumadin after acute coronary syndromes (the ASPECT‐2 study): a randomised controlled trial. Lancet. 2002;360:109‐113. 10.1016/S0140-6736(02)09409-6 12126819

[32] Hurlen M , Abdelnoor M , Smith P , Erikssen J , Arnesen H . Warfarin, aspirin, or both after myocardial infarction. N Engl J Med. 2002;347:969‐974. 10.1056/NEJMoa020496 12324552

[33] Parker CS , Chen Z , Price M , et al. Adherence to warfarin assessed by electronic pill caps, clinician assessment, and patient reports: results from the IN‐RANGE study. J Gen Intern Med. 2007;22:1254‐1259. 10.1007/s11606-007-0233-1 17587092 PMC2219760

[34] Kubitza D , Becka M , Wensing G , Voith B , Zuehlsdorf M . Safety, pharmacodynamics, and pharmacokinetics of BAY 59–7939–an oral, direct Factor Xa inhibitor–after multiple dosing in healthy male subjects. Eur J Clin Pharmacol. 2005;61:873‐880. 10.1007/s00228-005-0043-5 16328318

[35] Alexander JH , Lopes RD , James S , et al. Apixaban with antiplatelet therapy after acute coronary syndrome. N Engl J Med. 2011;365:699‐708. 10.1056/NEJMoa1105819 21780946

[36] Mega JL , Braunwald E , Mohanavelu S , et al. Rivaroxaban versus placebo in patients with acute coronary syndromes (ATLAS ACS‐TIMI 46): a randomised, double‐blind, phase II trial. Lancet. 2009;374:29‐38. 10.1016/S0140-6736(09)60738-8 19539361

[37] Kubitza D , Becka M , Voith B , Zuehlsdorf M , Wensing G . Safety, pharmacodynamics, and pharmacokinetics of single doses of BAY 59–7939, an oral, direct factor Xa inhibitor. Clin Pharmacol Ther. 2005;78:412‐421. 10.1016/j.clpt.2005.06.011 16198660

[38] Mega JL , Braunwald E , Wiviott SD , et al. Rivaroxaban in patients with a recent acute coronary syndrome. N Engl J Med. 2012;366:9‐19. 10.1056/NEJMoa1112277 22077192

[39] Bonaca MP , Bauersachs RM , Anand SS , et al. Rivaroxaban in peripheral artery disease after revascularization. N Engl J Med. 2020;382:1994‐2004. 10.1056/NEJMoa2000052 32222135

[40] Dangas GD , De Backer O , Windecker S . A controlled trial of rivaroxaban after transcatheter aortic‐valve replacement. Reply. N Engl J Med. 2020;383:e8. 10.1056/NEJMc2017351 32640145

[41] Wallentin L , Becker RC , Budaj A , et al. Ticagrelor versus clopidogrel in patients with acute coronary syndromes. N Engl J Med. 2009;361:1045‐1057. 10.1056/NEJMoa0904327 19717846

[42] Wiviott SD , Braunwald E , McCabe CH , et al. Prasugrel versus clopidogrel in patients with acute coronary syndromes. N Engl J Med. 2007;357:2001‐2015. 10.1056/NEJMoa0706482 17982182

[43] Tricoci P , Huang Z , Held C , et al. Thrombin‐receptor antagonist vorapaxar in acute coronary syndromes. N Engl J Med. 2012; 366: 20‐33. 10.1056/NEJMoa1109719 22077816

[44] Wang Y , Wang Y , Zhao X , et al. Clopidogrel with aspirin in acute minor stroke or transient ischemic attack. N Engl J Med. 2013;369:11‐19. 10.1056/NEJMoa1215340 23803136

[45] Johnston SC , Easton JD , Farrant M , et al. Clopidogrel and aspirin in acute ischemic stroke and high‐risk TIA. N Engl J Med. 2018;379:215‐225. 10.1056/NEJMoa1800410 29766750 PMC6193486

[46] Johnston SC , Amarenco P , Denison H , et al. Ticagrelor and aspirin or aspirin alone in acute ischemic stroke or TIA. N Engl J Med. 2020;383:207‐217. 10.1056/NEJMoa1916870 32668111

[47] Bath PM , Woodhouse LJ , Appleton JP , et al. Antiplatelet therapy with aspirin, clopidogrel, and dipyridamole versus clopidogrel alone or aspirin and dipyridamole in patients with acute cerebral ischaemia (TARDIS): a randomised, open‐label, phase 3 superiority trial. Lancet. 2018;391:850‐859. 10.1016/S0140-6736(17)32849-0 29274727 PMC5854459

[48] Cosentino F , Grant PJ , Aboyans V , et al. ESC guidelines on diabetes, pre‐diabetes, and cardiovascular diseases developed in collaboration with the EASD. Eur Heart J. 2019;2020(41):255‐323. 10.1093/eurheartj/ehz486 31497854

[49] Steg PG , Bhatt DL , Simon T , et al. Ticagrelor in patients with stable coronary disease and diabetes. N Engl J Med. 2019;381(14):1309‐1320. 10.1056/NEJMoa1908077 31475798

[50] Thun MJ , Henley SJ , Patrono C . Nonsteroidal anti‐inflammatory drugs as anticancer agents: mechanistic, pharmacologic, and clinical issues. J Natl Cancer Inst. 2002;94:252‐266. 10.1093/jnci/94.4.252 11854387

[51] Kune GA , Kune S , Watson LF . Colorectal cancer risk, chronic illnesses, operations, and medications: case control results from the Melbourne colorectal cancer study. Cancer Res. 1988;48:4399‐4404. 3390835

[52] FitzGerald GA , Patrono C . The coxibs, selective inhibitors of cyclooxygenase‐2. N Engl J Med. 2001;345:433‐442. 10.1056/NEJM200108093450607 11496855

[53] Kearney PM , Baigent C , Godwin J , Halls H , Emberson JR , Patrono C . Do selective cyclo‐oxygenase‐2 inhibitors and traditional non‐steroidal anti‐inflammatory drugs increase the risk of atherothrombosis? Meta‐analysis of randomised trials. BMJ. 2006;332:1302‐1308. 10.1136/bmj.332.7553.1302 16740558 PMC1473048

[54] Algra AM , Rothwell PM . Effects of regular aspirin on long‐term cancer incidence and metastasis: a systematic comparison of evidence from observational studies versus randomised trials. Lancet Oncol. 2012;13:518‐527. 10.1016/S1470-2045(12)70112-2 22440112

[55] Cole BF , Logan RF , Halabi S , et al. Aspirin for the chemoprevention of colorectal adenomas: meta‐analysis of the randomized trials. J Natl Cancer Inst. 2009;101:256‐266. 10.1093/jnci/djn485 19211452 PMC5975663

[56] Burn J , Bishop DT , Mecklin JP , et al. Effect of aspirin or resistant starch on colorectal neoplasia in the Lynch syndrome. N Engl J Med. 2008;359:2567‐2578. 10.1056/NEJMoa0801297 19073976

[57] Burn J , Sheth H , Elliott F , et al. Cancer prevention with aspirin in hereditary colorectal cancer (Lynch syndrome), 10‐year follow‐up and registry‐based 20‐year data in the CAPP2 study: a double‐blind, randomised, placebo‐controlled trial. Lancet. 2020;395:1855‐1863. 10.1016/S0140-6736(20)30366-4 32534647 PMC7294238

[58] Cook NR , Lee IM , Zhang SM , Moorthy MV , Buring JE . Alternate‐day, low‐dose aspirin and cancer risk: long‐term observational follow‐up of a randomized trial. Ann Intern Med. 2013;159:77‐85. 10.7326/0003-4819-159-2-201307160-00002 23856681 PMC3713531

[59] Rothwell PM , Fowkes FG , Belch JF , Ogawa H , Warlow CP , Meade TW . Effect of daily aspirin on long‐term risk of death due to cancer: analysis of individual patient data from randomised trials. Lancet. 2011;377:31‐41. 10.1016/S0140-6736(10)62110-1 21144578

[60] Rothwell PM , Price JF , Fowkes FG , et al. Short‐term effects of daily aspirin on cancer incidence, mortality, and non‐vascular death: analysis of the time course of risks and benefits in 51 randomised controlled trials. Lancet. 2012;379:1602‐1612. 10.1016/S0140-6736(11)61720-0 22440946

[61] Drew DA , Chan AT . Aspirin in the prevention of colorectal neoplasia. Annu Rev Med. 2021;72:415‐430. 10.1146/annurev-med-060319-120913 33035431 PMC7880546

[62] Rothwell PM , Wilson M , Price JF , Belch JF , Meade TW , Mehta Z . Effect of daily aspirin on risk of cancer metastasis: a study of incident cancers during randomised controlled trials. Lancet. 2012;379:1591‐1601. 10.1016/S0140-6736(12)60209-8 22440947

[63] Bibbins‐Domingo KUS . Aspirin use for the primary prevention of cardiovascular disease and colorectal cancer: U.S. Preventive Services Task Force recommendation statement. Ann Intern Med. 2016;164:836‐845. 10.7326/M16-0577 27064677

[64] Joharatnam‐Hogan N , Cafferty F , Hubner R , et al. Aspirin as an adjuvant treatment for cancer: feasibility results from the Add‐Aspirin randomised trial. Lancet Gastroenterol Hepatol. 2019;4:854‐862. 10.1016/S2468-1253(19)30289-4 31477558

[65] Soualy A , Deutsch D , Benallaoua M , et al. Effect of chemoprevention by low‐dose aspirin of new or recurrent colorectal adenomas in patients with Lynch syndrome (AAS‐Lynch): study protocol for a multicenter, double‐blind, placebo‐controlled randomized controlled trial. Trials. 2020;21:764. 10.1186/s13063-020-04674-8 32887653 PMC7487877

[66] Group. I‐SISoISC . Randomised trial of intravenous streptokinase, oral aspirin, both, or neither among 17,187 cases of suspected acute myocardial infarction: ISIS‐2. ISIS‐2 (Second International Study of Infarct Survival) Collaborative Group. Lancet. 1988;2:349‐360. 2899772

[67] Garcia Rodriguez LA . Variability in risk of gastrointestinal complications with different nonsteroidal anti‐inflammatory drugs. Am J Med. 1998; 104: 30S‐S34; discussion 41S‐2S. 10.1016/s0002-9343(97)00208-8 9572318

[68] Bhala N , Emberson J , Merhi A , et al. Vascular and upper gastrointestinal effects of non‐steroidal anti‐inflammatory drugs: meta‐analyses of individual participant data from randomised trials. Lancet. 2013;382:769‐779. 10.1016/S0140-6736(13)60900-9 23726390 PMC3778977

[69] Laureano MG , Barbui L , Chu T , Ferrari D , Siegal AD . Risks and benefits of antithrombotic therapy in polycythemia vera: a systematic review. Res Pract Thromb Haemost. 2020;4(Suppl 1):1046–7. Accessed February 19, 2021.32864555

[70] Karimi MHS . Silent brain ischemia in thalassemia patients: breaking the silence. Res Pract Thromb Haemost. 2020;4(Suppl 1):39. Accessed February 19, 2021.

[71] Karimi MHS , Eshghi P . Thromboembolism in thalassemia patients: is aspirin protective against brain ischemia over three years follow up in beta thalassemia patients? Res Pract Thromb Haemost. 2020;4(Suppl 1):38. Accessed February 19, 2021.

[72] Canzano PBM , Tortorici E , Cosentino N , et al.Disantangling the mechanisms behind the thrombotic complications of COVID‐19 patients: insights into platelet and endothelial activation. Res Pract Thromb Haemost. 2020;4(Suppl 2):9. Accessed February 19, 2021.31989078

[73] https://clinicaltrials.gov/ct2/show/NCT04363840. Accessed February 19, 2021

[74] https://www.pharmaceutical‐journal.com/news‐and‐analysis/news/aspirin‐added‐to‐recovery‐covid‐19‐trial‐mps‐told/20208522.article. Accessed January 30, 2021

[75] Mourikis PZS , Dannenberg L , Helten C , et al. Aspirin antiplatelet effects are associated with body weight. Res Pract Thromb Haemost. 2020;4(Suppl 1):28. Accessed February 19, 2021. 10.1016/j.vph.2019.106635 31862488

[76] Porro BDM , Veglia F , Giroli M , et al. Systemic aspirin esterification in subjects with cardiovascular diseases: impact of body size. Res Pract Thromb Haemost. 2020;4(Suppl 1):34. Accessed February 19, 2021.

[77] Rocca B , Tosetto A , Betti S , et al. A randomized double‐blind trial of 3 aspirin regimens to optimize antiplatelet therapy in essential thrombocythemia. Blood. 2020;136:171‐182. 10.1182/blood.2019004596 32266380

[78] Cavalca V , Rocca B , Veglia F , et al. On‐pump cardiac surgery enhances platelet renewal and impairs aspirin pharmacodynamics: effects of improved dosing regimens. Clin Pharmacol Ther. 2017;102:849‐858. 10.1002/cpt.702 28379623

[79] Hoffmann TR , Kirchhoff E , Yilmaz E , et al.Postoperative dual antiplatelet therapy is infuenced by platelet regeneration. Res Pract Thromb Haemost. 2020;4(Suppl 1):10. Accessed February 19, 2021.

[80] Tanaka H , Horioka K , Yamazaki K , Ogawa K . Amelioration of the pathologic changes in the hepatocyte‐SpeciBRAFV600E mutated mice by administration of aspirin. Res Pract Thromb Haemost. 2020;4(Suppl 1):1044. Accessed February 19, 2021.

[81] Wong PC , Dilger A , Wexler R , Ewing W , Gordon D , Luettgen J . Small‐molecule factor XIa inhibitor, BMS‐986177/JNJ‐70033093, prevents and treats arterial thrombosis in rabbits at doses that preserve hemostasis. Res Pract Thromb Haemost. 2020;4(Suppl 1):52. Accessed February 19, 2021.

[82] Collet JP , Thiele H , Barbato E , et al. ESC Guidelines for the management of acute coronary syndromes in patients presenting without persistent ST‐segment elevation. Eur Heart J. 2021;42(14):1289–1367. 10.1093/eurheartj/ehaa575 32860058

[83] Knuuti J , Wijns W , Saraste A , et al. ESC guidelines for the diagnosis and management of chronic coronary syndromes. Eur Heart J. 2020;41(3):407–477. 10.1093/eurheartj/ehz425 31504439

[84] Galiuto L , Patrono C . Aspirin monotherapy vs. DAPT after TAVI: is less more? Comment on the POPular TAVI trial. Eur Heart J. 2020;41:4301‐4302. 10.1093/eurheartj/ehaa800 33099597

[85] Brouwer J , Nijenhuis VJ , Delewi R et al. Aspirin with or without clopidogrel after transcatheter aortic‐valve implantation. N Engl J Med. 2020;383(15):1447‐1457. 10.1056/NEJMoa2017815 32865376

